# Syndecan-1 induction in lung microenvironment supports the establishment of breast tumor metastases

**DOI:** 10.1186/s13058-018-0995-x

**Published:** 2018-07-05

**Authors:** Colleen Chute, Xinhai Yang, Kristy Meyer, Ning Yang, Keelin O’Neil, Ildiko Kasza, Kevin Eliceiri, Caroline Alexander, Andreas Friedl

**Affiliations:** 1 Department of Pathology and Laboratory Medicine, University of Wisconsin-Madison, 6051 WIMR, MC-2275, 1111 Highland Avenue, Madison, WI 53705 USA; 2 Pathology and Laboratory Medicine Service, William S. Middleton Memorial Veterans Hospital, Department of Veterans Affairs Medical Center, Madison, WI USA; 3 University of Wisconsin Carbone Cancer Center, Madison, WI USA; 4 Laboratory for Optical and Computational Instrumentation, University of Wisconsin-Madison, Madison, WI USA; 5 Morgridge Institute for Research, University of Wisconsin-Madison, Madison, WI USA; 6 Department of Oncology, University of Wisconsin-Madison, Madison, WI USA

**Keywords:** Breast cancer, Proteoglycans, Extracellular matrix, Metastasis, Syndecan, Tumor microenvironment

## Abstract

**Background:**

Syndecan-1 (Sdc1), a cell surface heparan sulfate proteoglycan normally expressed primarily by epithelia and plasma cells, is aberrantly induced in stromal fibroblasts of breast carcinomas. Stromal fibroblast-derived Sdc1 participates in paracrine growth stimulation of breast carcinoma cells and orchestrates stromal extracellular matrix fiber alignment, thereby creating a migration and invasion-permissive microenvironment. Here, we specifically tested the role of stromal Sdc1 in metastasis.

**Methods:**

The metastatic potential of the aggressive mouse mammary carcinoma cell lines, 4T1 and E0776, was tested in wild-type and genetically Sdc1-deficient host animals. Metastatic lesions were characterized by immunohistochemical analysis.

**Results:**

After orthotopic inoculation, the lung metastatic burden was reduced in Sdc1−/− animals by 97% and more than 99%, in BALB/cJ and C57BL/6 animals, respectively. The difference in metastatic efficiency was maintained when the tumor cells were injected into the tail vein, suggesting that host Sdc1 exerts its effect during later stages of the metastatic cascade. Co-localization studies identified Sdc1 expression in stromal fibroblasts within the metastatic microenvironment and in normal airway epithelial cells but not in other cells (endothelial cells, α-smooth muscle actin positive cells, leucocytes, macrophages). The Ki67 proliferation index and the rate of apoptosis of the metastatic tumor cells were diminished in Sdc1−/− vs. Sdc1+/+ animals, and leucocyte density was indistinguishable. Sdc1-mediated metastatic efficiency was abolished when the animals were housed at a thermoneutral ambient temperature of 31 °C, suggesting that the host Sdc1 effect on metastasis requires mild cold stress.

**Conclusions:**

In summary, Sdc1 is induced in the lung microenvironment after mammary carcinoma cell dissemination and promotes outgrowth of metastases in a temperature-dependent manner.

**Electronic supplementary material:**

The online version of this article (10.1186/s13058-018-0995-x) contains supplementary material, which is available to authorized users.

## Background

The fate of patients with breast carcinoma is determined by distant organ metastasis rather than local disease. Life-threatening metastatic disease is the end-result of a cascade of events that begins with local invasion at the primary site and includes intravasation (into blood or lymphatic vessels), survival of tumor cells in the blood stream, extravasation at the distant site and outgrowth of metastatic lesions. Recent experimental evidence suggests that the initial steps of the metastatic cascade including intravasation occur relatively early and that the later events like extravasation and outgrowth may be the rate-limiting steps [1]. Although progress has been made in uncovering the biology of some aspects of metastatic spread, little is known about the mechanisms that govern adaptation of disseminated tumor cells to the environment at the distant site and determine whether tumor cells remain dormant or actively proliferate. It is becoming increasingly clear, however, that complex reciprocal interactions between disseminated tumor cells and cells in the local microenvironment (i.e. the metastatic niche) play a crucial role [2].

Syndecan-1 (Sdc1; CD138) belongs to a four-member family of transmembrane heparan sulfate proteoglycans (HSPGs) with roles in cell signaling and adhesion [3]. Sdc1 is primarily expressed by plasma cells and epithelia, including their malignant counterparts. During development, Sdc1 expression is transiently induced in the mesenchyme and the molecule participates in paracrine epithelial-stromal interactions [4]. This mesenchymal induction is recapitulated during malignant progression, when Sdc1 expression is observed in stromal fibroblasts in a variety of carcinoma types, including carcinoma of the breast [5, 6]. Little is known about the mechanisms of Sdc1 induction in fibroblasts. Induction in mesenchymal cells has been linked to transcriptional regulation by members of the fibroblast growth factor family and extracellular matrix (ECM) constituents [7–9].

Via its heparan sulfate (HS) chains, Sdc1 engages HS-binding ligands including growth factors and many ECM molecules - a property it shares with other HSPGs [10]. Sdc1 core protein-specific binding interactions have been observed between its ectodomain and both integrin cell adhesion receptor subunits and receptor tyrosine kinases [11]. Thus, Sdc1 can act as a cell surface docking station that complexes integrins and receptor tyrosine kinases (RTKs), thereby regulating cell growth and migration. Mice globally deficient in Sdc1 have a surprisingly subtle phenotype with a slight reduction in size and weight noted as the sole abnormality [12]. When the animals are challenged, however, some defects emerge. Sdc1-knockout animals display impaired inflammatory responses, have altered vascular and endothelial cell biology and reduced tumorigenesis [12–15]. Sdc1 deficiency also affects lipid metabolism and reduces tolerance to cold temperatures [16, 17]. The molecular pathways involved in these impaired responses to external and intrinsic challenges are largely unknown.

In breast cancer, Sdc1 generally acts as a promoter of tumor growth and progression via multiple mechanisms of action. Sdc1 overexpression in human breast carcinoma correlates with a proliferative state and poor prognosis [18–20]. Mice lacking Sdc1 are relatively resistant to Wnt-induced tumorigenesis, demonstrating that Sdc1 is required for efficient tumorigenesis in this model [12]. Sdc1 modulates tumor progression not only by cell autonomous but also by cell non-autonomous mechanisms. The induction of Sdc1 expression in stromal fibroblasts triggers a reciprocal paracrine signaling loop that stimulates mammary tumor growth in vitro and in vivo [6, 21, 22]. Sdc1-expressing stromal fibroblasts also produce an altered ECM that is characterized by parallel, aligned fibronectin and collagen fibers, which is permissive to carcinoma cell migration and invasion and thus has the potential to promote carcinoma spread and metastasis [23]. Collectively, these findings indicate that Sdc1 can stimulate breast tumor progression at many levels.

The goal of the present study was to determine whether host Sdc1 plays a role in mammary carcinoma metastasis. We showed in two mouse strains that the ability of highly aggressive mouse mammary tumor cells to metastasize to the lungs is diminished in mice genetically deficient in Sdc1. The requirement of host Sdc1 for efficient metastasis is observed both after orthotopic (fat pad) and tail vein injection, which suggests that Sdc1 exerts its effect during the later steps of the metastatic cascade; likely during metastatic outgrowth. Elevating the ambient housing temperature to thermo-neutral conditions reduces metastatic efficiency in the wild-type animals to the level seen in the knockout mice suggesting that the Sdc1-dependent mechanism affecting metastasis is regulated by the thermogenic response.

## Methods

### Cells, tumor cell inoculations and scoring of metastases

The 4T1 mouse mammary tumor cells were obtained from American Type Culture Collection (ATCC) (CRL-2539) and were cultured in Roswell Park Memorial Institute (RPMI-1640) medium supplemented with 10% fetal bovine serum (FBS), 2 mM L-glutamine and penicillin/streptomycin at 37 °C in a humidified atmosphere containing 5% CO_2_. E0771 cells were purchased from CH3 Biosystems (Amhurst, NY, USA) and cultured in RPMI-1640 medium supplemented with 10 mmol/L HEPES and 10% FBS.

For fat pad injections, mice were anesthetized with isoflurane, and 1 × 10^7^ cells in 10 μL serum-free DMEM were injected into the exposed, intact left 4th mammary fat pad as described by Miller [24]. Tumors were allowed to grow for 30 days and then mice were humanely killed. For tail vein injections, mice were anesthetized with isoflurane and 1 × 10^5^ tumor cells in 100 μL serum-free DMEM were injected through the tail-vein. After 15 days the mice were humanely killed.

Upon completion of in vivo studies, tissues were fixed for 12–18 h in 10% buffered formalin (Fisher Scientific, Waltham, MA, USA) and then processed and paraffin embedded. Hematoxylin and eosin (H&E)-stained slides were either scanned with an Aperio whole slide scanner (Leica Biosystems) or imaged by stitching individually acquired images in Adobe Photoshop. The image files showing sections of whole lungs were carefully examined and metastatic lesions were circled with an Intuos input device (Wacom) and analyzed using a combination of Photoshop (Adobe) and ImageJ (https://imagej.nih.gov/ij/). The number of lesions per mouse, the area of each lesion and the area of total lung tissue were recorded. Tumor burden per mouse was defined as area of lung tissue occupied by metastases divided by total area of lung.

### Antibodies, reagents and histological analyses

The antibodies used for immunolabeling are listed in Table 1. Mouse tissue sections were deparaffinized and for antigen retrieval, sections were boiled with citrate buffer (pH 6.0, with 0.05% Tween 20) for 30 min. In preparation for fibroblast antibody (ER-TR7) labeling, sections of mouse tissues were incubated with proteinase K working solution (20 μg/mL in TE buffer, pH 8.0) for 15 min at 37 °C. After incubation with the primary antibody (overnight at 4 °C for CD4 and CD8; 1 h at room temperature for all others) and extensive washes, horseradish peroxidase chromogenic (Ventana) or TSA Plus fluorescence detection kits (Perkin Elmer) were applied following the manufacturers’ instructions. Nuclei were counterstained with hematoxylin, 4′,6-diamidino-2-phenylindole (DAPI) or Hoechst 33,342 as appropriate. CD4 and CD8 positive T cells were visualized in mouse lung sections by manual immunolabeling using the ImmPRESS polymer detection system and diaminobenzidine (DAB) substrate.

**Table 1:** Antibodies used for immunolabeling

Antigen	Dilution	Specificity	Catalog number	Company/source
CD31	1:400	Goat anti-mouse	AF3628	R&D Systems, Minneapolis, MN, USA
Ki67	1:1000	Mouse anti-human, clone MIB-1	M7240	Dako, Santa Clara, CA, USA
Ki67	1:200	Rabbit anti-mouse, clone D3B5	12,202	Cell Signaling Technology, Danvers, MA, USA
αSMA	1:500	Rabbit anti-mouse	ab5694	Abcam, Cambridge, MA, USA
F4/80	1:400	Rabbit anti-mouse	ab100790	Abcam
ER-TR7	1:200	Rat anti-mouse	ab51824	Abcam
CD45	1:200	Rabbit anti-mouse	ab10558	Abcam
CD68	1:200	Rat anti-mouse	MCA1957T	BioRad
Cleaved Caspase-3	1:200	Rabbit anti-mouse, D175	9661	Cell Signaling Technology
Sdc-1	1:200	Rat anti-mouse	NA	gift from Dr. Rapraeger
Sdc-1	1:100	Mouse anti-human, clone B-B4	MCA681H	Serotec
Vimentin	predilute	Mouse anti-human	790–2917	Ventana Medical Systems, Tucson, AZ, USA
CD4	1:200	Rabbit anti-mouse	ab221775	Abcam
CD8	1:750	Rabbit anti-mouse	ab209775	Abcam

For the analysis of immunohistochemically labeled slides, at least five images per sample were captured using a brightfield or epifluorescence microscope depending on the label. The signal was quantified using a combination of Photoshop and the National Institutes of Health (NIH) ImageJ software [25]. Lymphocyte densities were determined in the two smallest and the two largest metastases per lung. CD4+ and CD8+ lymphocyte densities were expressed as cells per 1 × 10^6^ pixel (megapixel) on images taken at consistent magnification and resolution.

Tissue sections of breast carcinoma metastases to the lung from seven patients were analyzed by dual-labeling for Sdc1 and the mesenchymal/stromal marker vimentin. After deparaffinization and heat-induced antigen retrieval (EDTA, pH 8.5, 95–100 °C for 44 min), sections were incubated with anti-Sdc1 antibody (8 min, 37 °C). After rinsing, the UltraMap mouse HRP polymer kit (Ventana, Roche, catalog number 760–4313) was applied following the manufacturer’s instructions. After denaturing and rinsing, a second round of epitope retrieval under the same conditions was applied and the sections were incubated with prediluted anti-vimentin antibody (16 min, 37 °C). After rinsing, HQ mouse polymer (Roche, 760–4814) was applied for 8 min at 37 °C followed by anti-HQ HRP solution (Roche, 760–4820; 8 min, 37 °C) and the Discovery purple detection kit (Roche 760–229).

### Second harmonic generation microscopy and collagen fiber analysis

H&E-stained histology slides were placed onto an optical workstation built around a Nikon Eclipse TE300 (Nikon, Tokyo, Japan), with a Ti:Sapphire laser (Spectra-Physics-Millennium/Tsunami, Mountain View CA, USA) excitation source tuned to 890 nm, focused with a × 20 Nikon Plan Apo lens (Nikon, Tokyo, Japan), filtered with a 445 nm narrow band pass filter (TFI Technologies, Greenfield MA, USA) and back-scattered second harmonic generation (SHG) signal collected with an H7422P-40 detector (Hamamatsu, Japan) and WiscScan acquisition software developed at the Laboratory for Optical and Computational Instrumentation (LOCI), University of Wisconsin, Madison, WI, USA).

Collagen fiber angles relative to the tumor boundary were analyzed using CTFIRE and the MATLAB-based CurveAlign software developed by the LOCI (http://loci.wisc.edu/software/curvealign) [26]. Intensity of the SHG images was analyzed using the NIH ImageJ Software (as described above).

### Animals

The generation of Sdc1−/− mice has been described previously [12]. Mice were housed at room temperature (20–23 °C unless otherwise specified) and maintained on a 12-h light and dark cycle with free access to water and food. For all tumor experiments, 6–8 week old female mice were used. For experiments using thermo-neutral conditions, mice were individually caged and housed at 31 °C (± 1 °C) for 2 weeks in a controlled environment prior to cell inoculation and were monitored daily.

### Statistics

Data are expressed as mean +/− standard error of the mean. Statistical tests were performed using MStat, which is JAVA-based software written at the University of Wisconsin-Madison (http://mcardle.wisc.edu/mstat/) or Prism 7 software (Graphpad). The non-parametric Wilcoxon rank sum, Mann-Whitney or Kruskal-Wallis tests were used unless stated otherwise and *p* values less than 0.05 were deemed to be significant.

## Results

### Host Sdc1 is required for efficient metastasis of mammary carcinoma cells to the lungs

**Fig. 1:**
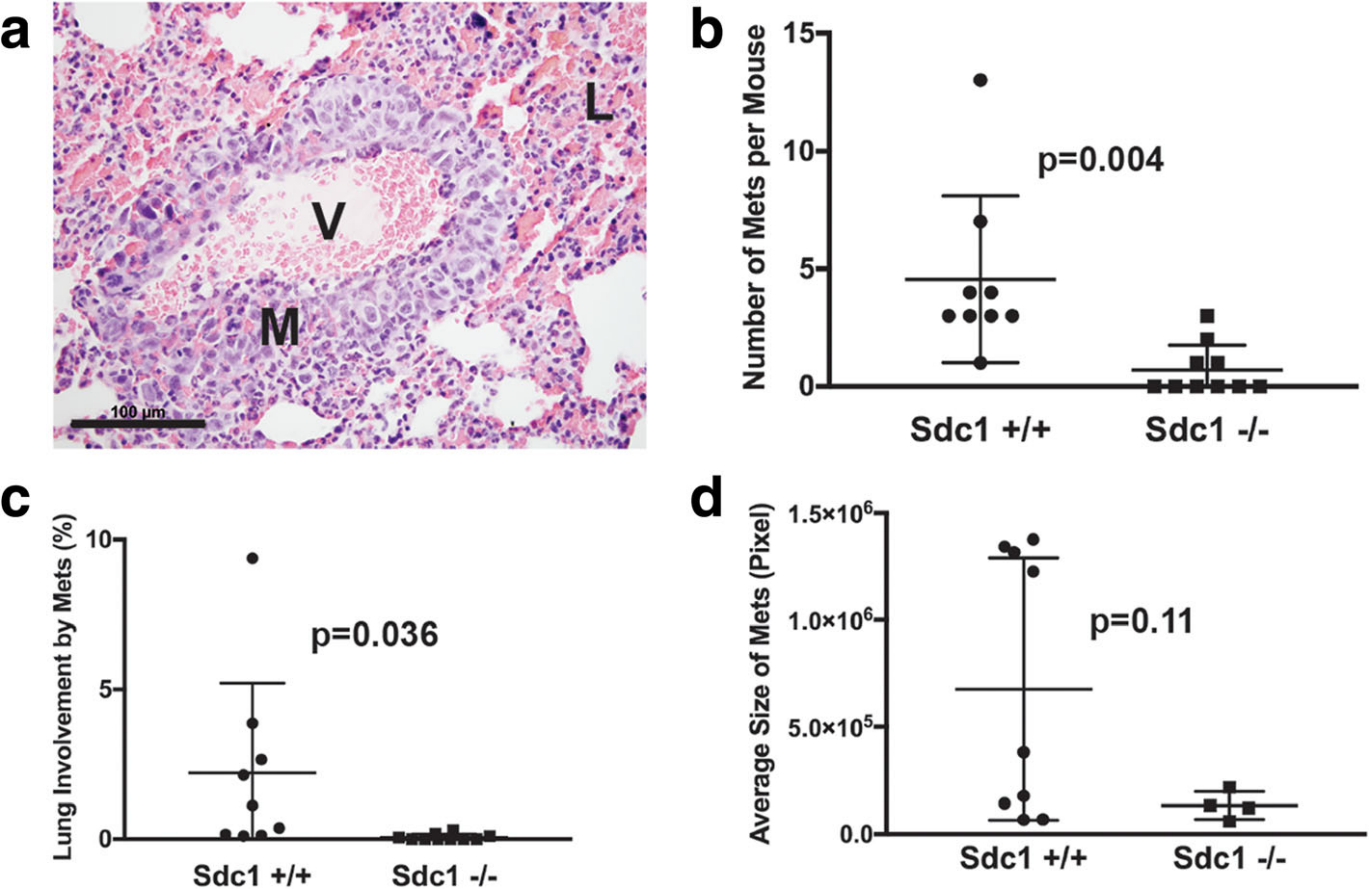
Effect of host syndecan-1 (Sdc1) on metastatic efficiency of 4T1 mouse mammary carcinoma cells. The 4T1 tumor cells (1 × 10^7^ cells in 10 μL serum-free DMEM) were injected into the exposed 4th mammary gland as described in “Methods” and mice were sacrificed after 30 days. **a** Small, early metastasis, cuffing blood vessel in lung (M, metastasis; V, vessel; L, lung parenchyma; original magnification × 400; scale bar indicates 100 μm). **b** Number of metastatic lesions per mouse. Metastases were counted on single histologic sections of both lungs. Bars indicate mean +/− standard deviation. **c** Metastatic tumor burden expressed as percent lung tissue occupied by metastatic lesions (see “Methods” for details). **d** Average area of metastatic lesions expressed in pixels as measured on histologic sections

Both carcinoma-cell-associated and stromal Sdc1 can promote breast carcinoma growth [6, 20, 22] but the importance of this proteoglycan in breast carcinoma progression and metastatic spread is less clear. To determine the role of host Sdc1 in metastasis, we inoculated highly metastatic 4T1 mouse mammary carcinoma cells into the mammary fat pads of syngeneic BALB/cJ wild-type or genetically Sdc1-deficient mice. After 30 days, the number of metastases per mouse (Fig. 1a, b; *p* = 0.004) and the metastatic burden, defined as percent of lung tissue occupied by metastases (Fig. 1c; *p* = 0.036) were significantly reduced in Sdc1−/− compared to Sdc1+/+ animals. The average size of metastatic lesions was not significantly different between Sdc1−/− and Sdc1+/+ mice (Fig. 1d; *p* = 0.11) - presumably because of the small number of metastases in the Sdc1−/− mice and the high variability of the size of metastatic lesions in the Sdc1+/+ mice.

Since tumor behavior can be highly dependent on mouse strain variations [27] and because rate-limiting steps of metastasis may differ between carcinoma cell lines, we tested the effect of host Sdc1 in a different mouse mammary tumor metastasis model. The E0771 mammary tumor cells metastasize primarily to the lungs of C57BL/6 mice, similar to 4T1 cells in BALB/cJ animals [28]. The results with C57BL/6 animals mirrored our observations in BALB/cJ mice, as the number of metastases per mouse (Additional file 1: Figure S1A, B; *p* = 0.013) and the metastatic burden (Additional file 1: Figure S1C; *p* = 0.038) were significantly reduced in Sdc1−/− compared to Sdc1+/+ animals. The average size of metastatic lesions was not significantly reduced in Sdc1−/− compared to Sdc1+/+ mice (Additional file 1: Figure S1D; *p* = 0.37). This result shows that host Sdc1 affects metastatic efficiency independent of mouse strain and cell line and implicates this proteoglycan as a regulator of metastasis.

### Host Sdc1 does not significantly affect growth or microenvironmental characteristics of primary tumors

In the fat pad inoculation model, Sdc1 may affect any step of the metastatic cascade - from local invasion and intravasation, to extravasation and metastatic outgrowth. Previous work by our group and others has shown that Sdc1 is induced in stromal fibroblasts of breast carcinomas in both humans and in mice and that stromal Sdc1 can stimulate tumor growth and angiogenesis and create an invasion-permissive microenvironment [6, 21–23]. However, in this model, the average weight of mammary fat pad tumors grown in Sdc1+/+ or Sdc1−/− mice did not differ significantly (Additional file 2: Figure S2A). The absence of host Sdc1 did not affect the Ki67 proliferation index in the primary carcinoma cells, indicating that host Sdc1 does not significantly stimulate 4T1 carcinoma cell proliferation in the primary tumor site (Additional file 2: Figure S2B).

Since stromal Sdc1 has previously been shown to stimulate angiogenesis [22], we studied the tumor vasculature by labeling primary tumor sections with an antibody to the endothelial cell marker CD31. However, in this model, no difference in microvessel density was detected between tumors arising in Sdc1+/+ or Sdc1−/− mice (Additional file 2: Figure S2C). Myofibroblasts or carcinoma-associated fibroblasts (CAF) and macrophages not only stimulate tumor growth but also modulate the metastatic behavior of carcinoma cells [29, 30]. Therefore, we examined whether host Sdc1 affects these cellular constituents of the local tumor microenvironment. Alpha smooth actin (αSMA)-positive cells were arranged in a pattern similar to CD31-positive cells, suggesting that most intratumoral αSMA-positive cells are vascular smooth muscle cells or pericytes. The density of intratumoral αSMA-positive cells was similar in Sdc1+/+ and Sdc1−/− host animals (Additional file 2: Figure S2D). We were also unable to detect a difference in the density of intratumoral macrophages (Additional file 2: Figure S2E).

Given the role of stromal Sdc1 in ECM fiber assembly in vitro and in vivo, we measured the amount of collagen in the 4T1 mammary tumors by SHG imaging and collagen alignment by computer-assisted analysis of the SHG images [26, 31]. Collagen fibers were found primarily in the periphery of the tumors aggregating into a “pseudo-capsule” (Additional file 2: Figure S2F). There was no significant difference in the amount of collagen (SHG signal intensity/area; not shown) nor in the mean angles between collagen fibers and the tumor boundary when comparing Sdc1+/+ and Sdc1−/− mice (Additional file 2: Figure S2F). In summary, apart from insignificantly smaller tumor size in Sdc1−/− animals, no Sdc1-related differences were identified in the primary 4T1 mammary fat pad tumors.

### Host Sdc1 is important during later stages of mammary carcinoma metastasis

**Fig. 2:**
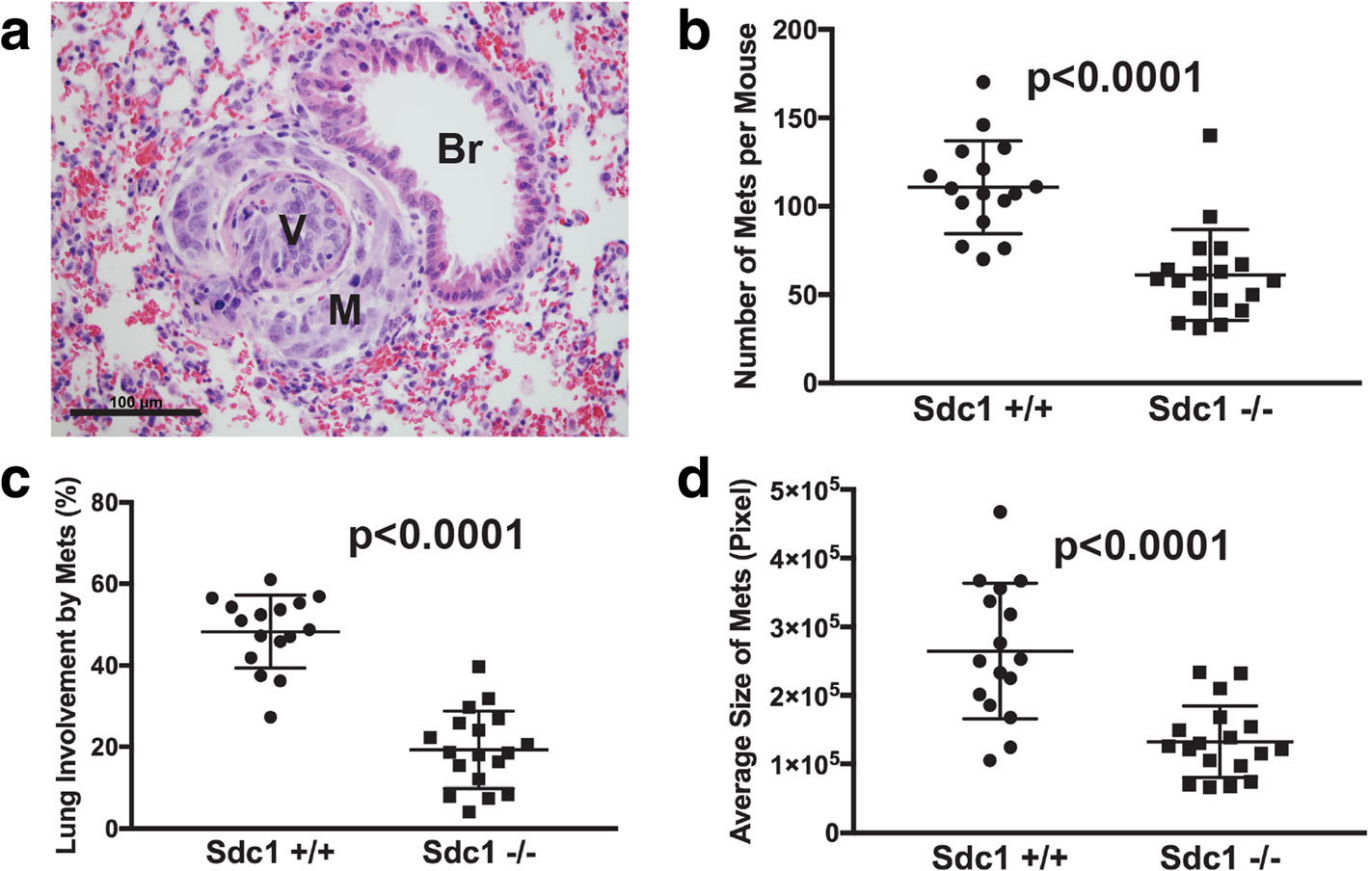
Effect of host syndecan-1 (Sdc1) on later stages of the metastatic cascade. The 4T1 tumor cells (1 × 10^5^ tumor cells in 100 μL serum-free DMEM) were injected into the tail vein and mice were sacrificed 15 days later. **a** Small, early metastasis, with carcinoma cells invading through lung vessel wall and surrounding vessel (M, metastasis; V, vessel; Br, bronchiole; original magnification × 400; scale bar indicates 100 μm). **b** Number of metastatic lesions per mouse. Metastases were counted on single histologic sections of both lungs. **c** Metastatic tumor burden expressed as percent lung tissue occupied by metastatic lesions. **d** Average area of metastatic lesions expressed in pixels as measured on histologic sections

Since host Sdc1-dependent differences in metastatic efficiency could not be attributed to differences in the primary tumors, we focused our attention to later stages of the metastatic cascade. After tail vein injection of tumor cells - an inoculation method that bypasses the early stages (local invasion, intravasation) of metastatic spread - host Sdc1-dependent differences in metastatic efficiency were maintained (Fig. 2). Specifically, the number of metastases (Fig. 2b), metastatic burden (Fig. 2c) and average size of metastatic lesions (Fig. 2d) were significantly reduced in Sdc1−/− mice compared to Sdc1+/+ animals (*p* < 0.0001 for all comparisons). This observation suggests that host Sdc1 is important at the distant organ site for any or all of the events occurring late during metastatic spread: extravasation, tumor cell survival, escape from dormancy or outgrowth of disseminated tumor cells; however, it does not exclude the possibility that host Sdc1 in the primary tumor also contributes to metastatic efficiency. Similarly, in the C57BL/6 model, the number of metastatic lesions (Additional file 3: Figure S3A, B; *p* = 0.045) and the metastatic burden (Additional file 3: Figure S3C; *p* = 0.037) were lower in Sdc1−/− mice, and the average size of the metastatic lesions was not significantly different (Additional file 3: Figure S3D).

### Sdc1 is induced in stromal fibroblasts of mammary carcinoma lung metastasis

**Fig. 3:**
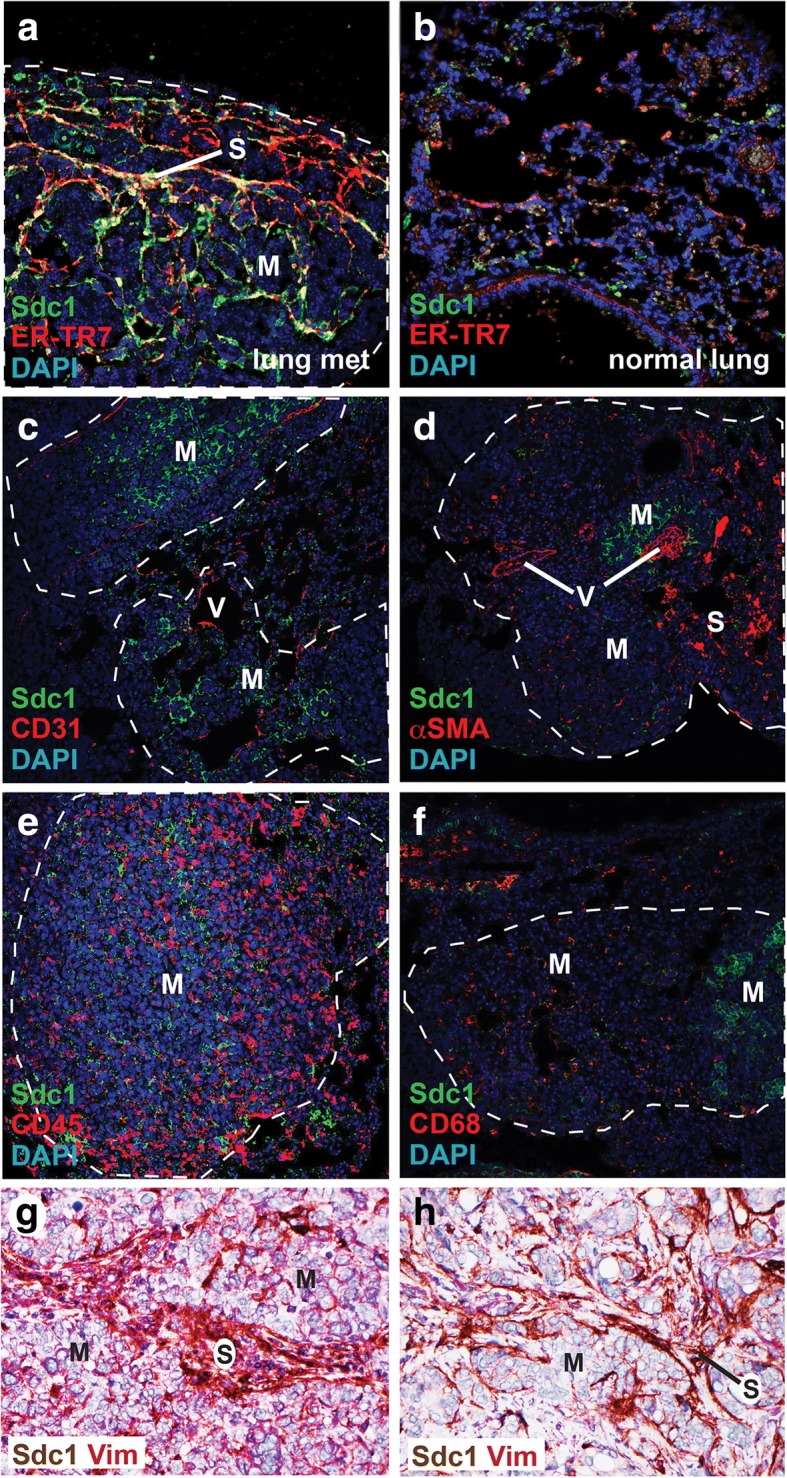
Syndecan-1 (Sdc1) expression in the metastatic microenvironment. Tissue sections are from tail-vein-injected 4T1 cells in Sdc1+/+ mouse (**a**-**f**) or human (**g**, **h**) mammary carcinoma lung metastases unless stated otherwise and were labeled with antibodies to Sdc1 and cell lineage markers as indicated. **a** Sdc1 (green) and fibroblast marker ER-TR7 (red). **b** Normal lung tissue labeled for Sdc1 (green) and fibroblast marker ER-TR7 (red). **c** Sdc1 (green) and endothelial cell marker CD31 (red). **d**) Sdc1 (green) and myofibroblast/smooth muscle/pericyte marker alpha smooth muscle actin (αSMA) (red). **e** Sdc1 (green) and leucocyte marker CD45 (red). **f** Sdc1 (green) and macrophage marker CD68 (red). **g** Human breast cancer metastasis to the lung labeled for Sdc1 (brown) and mesenchymal marker vimentin (Vim, magenta). Original magnification approximately × 400 for all images. Metastatic lesions are outlined with dashed lines. M, metastasis; S, stroma; V, vessel; DAPI, 4′,6-diamidino-2-phenylindole

Sdc1 expression is induced in stromal fibroblasts of primary mammary carcinomas in mice and in humans [5, 6]. In view of our finding that host Sdc1 plays a role in the metastatic niche, we examined whether Sdc1 is induced in stromal fibroblasts in lung metastatic lesions. The stromal compartment is relatively sparse in 4T1-derived lung metastases, yet we observed co-localization between Sdc1 and fibroblasts (identified with antibodies to the fibroblast marker ER-TR7) (Fig. 3a). Sdc1 was absent from (sparse) fibroblasts in normal lung tissue (Fig. 3b). Sdc1 did not co-localize with CD31-positive endothelial cells, nor with αSMA-positive cells, CD45-positive leukocytes or CD68-positive macrophages (Fig. 3c–f). Similar to the primary tumors, αSMA-positive cells were found primarily in a vascular pattern, consistent with the distribution of vascular smooth muscle cells or pericytes rather than myofibroblasts. Sdc1 is also present in airway epithelial cells (not shown). Tumor cells express Sdc1 constitutively and as allografts, independent of the mouse host genotype. Biopsy samples from a small collective of patients with breast carcinoma metastases to the lung showed a variable amount of stroma in the metastatic lesions. Stromal fibroblasts in the metastases expressed Sdc1 (Fig. 3g, h), mirroring Sdc1 induction in primary human breast carcinomas. As expected, Sdc1 was also expressed by carcinoma cells in mouse and human samples (Fig. 3c-h).

### Loss of host Sdc1 decreases proliferation and apoptosis in metastatic mammary carcinoma cells but does not affect leucocyte density

**Fig. 4:**
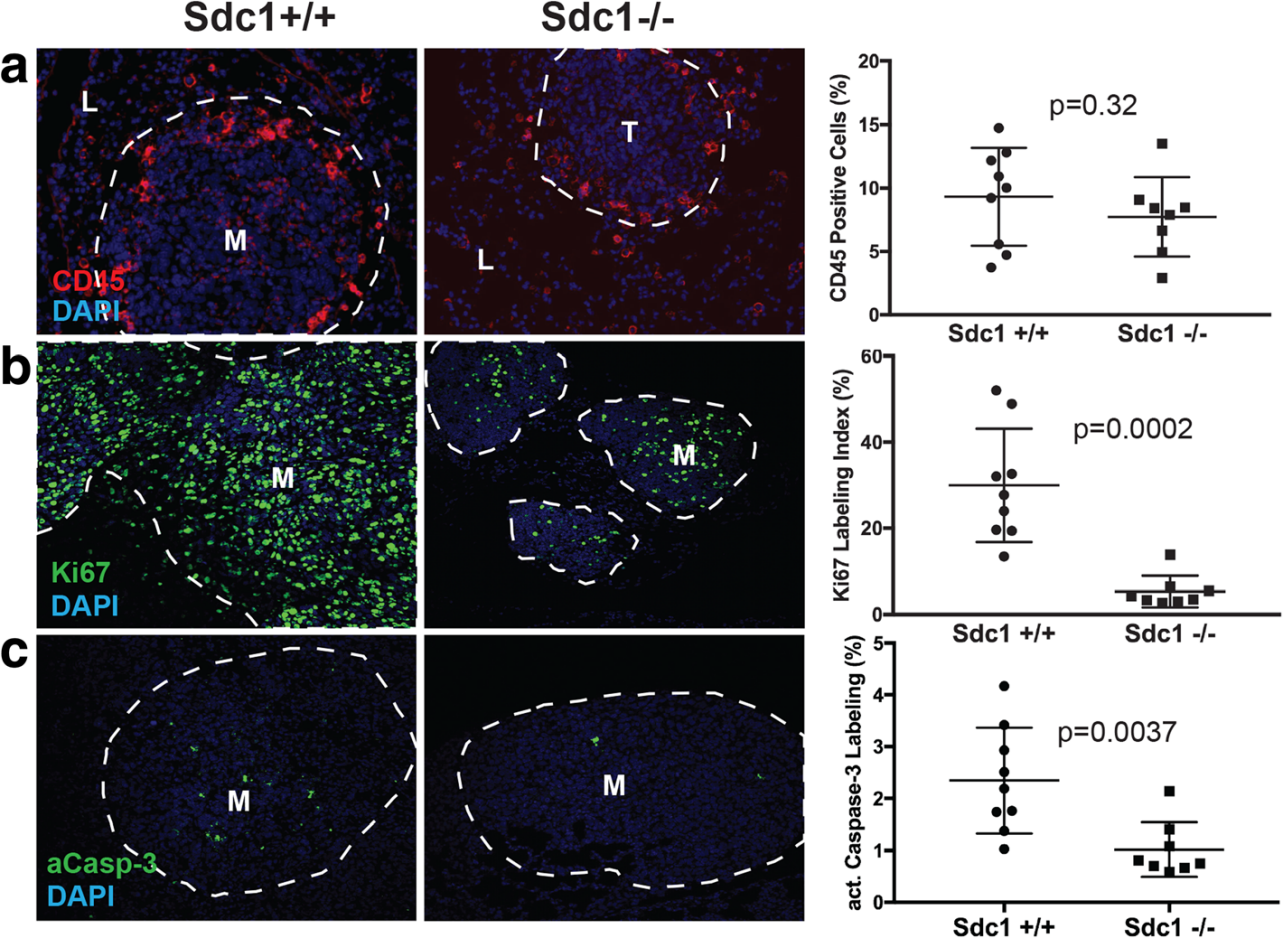
Characterization of the metastatic microenvironment and metastatic carcinoma cell proliferation/death. Lung metastases arising in Sdc1 +/+ and Sdc1−/− mice after tail vein injection of 4T1 cells were analyzed by immunofluorescence or second harmonic generation microscopy and the signal was quantified using ImageJ. **a** Leukocyte marker CD45 (red) **b** Proliferation marker Ki67 (green). **c** Apoptosis marker active caspase 3 (aCasp 3) (green). Original magnification approximately × 400 for all images. Metastatic lesions are outlined with dashed lines. Abbreviations: M, metastasis; L, lung parenchyma; DAPI, 4′,6-diamidino-2-phenylindole

As a first step towards determining the mechanism of action of Sdc1 at the lung metastatic site, we further characterized 4T1 metastatic lesions in the lungs. Inflammation is considered a driver of tumor progression and different leucocyte populations have been implicated in metastasis. Previous studies have shown that loss of Sdc1 leads to a pro-inflammatory phenotype in the endothelium and enhanced leucocyte recruitment [32, 33]. Also, neutrophils support lung colonization in several mammary tumor models [34] and certain macrophage sub-populations promote mouse mammary tumor metastasis by stimulating extravasation and growth [35, 36]. In our study, 4T1 lung metastases contained CD45-positive leucocytes primarily in the lesion periphery (Fig. 4a). Leucocyte density was indistinguishable between metastases arising in Sdc1+/+ vs. Sdc1 −/− mice, and therefore it is unlikely that an Sdc1-mediated increase or decrease in inflammation/immune cell infiltration is responsible for differences in metastatic efficiency.

Stromal Sdc1 has been shown to promote breast carcinoma cell proliferation via paracrine pathways [6, 21, 22, 37]. Therefore, Sdc1-dependent metastatic efficiency may be due to the stimulation of carcinoma cell proliferation by stromal cell-derived Sdc1. The Ki67 proliferation index in metastatic carcinoma cells was significantly (*p* = 0.0002) decreased by 86% in Sdc1−/− mice compared to their Sdc1+/+ counterparts (Fig. 4b). The proportion of apoptotic carcinoma cells - identified by active caspase 3 expression - was also lower in Sdc1−/− animals by 64% (Fig. 4c). Overall, these data are consistent with Sdc1 in the metastatic microenvironment stimulating the proliferation of carcinoma cells.

### The effect of host Sdc1 on metastatic efficiency is abolished in thermo-neutral conditions

**Fig. 5:**
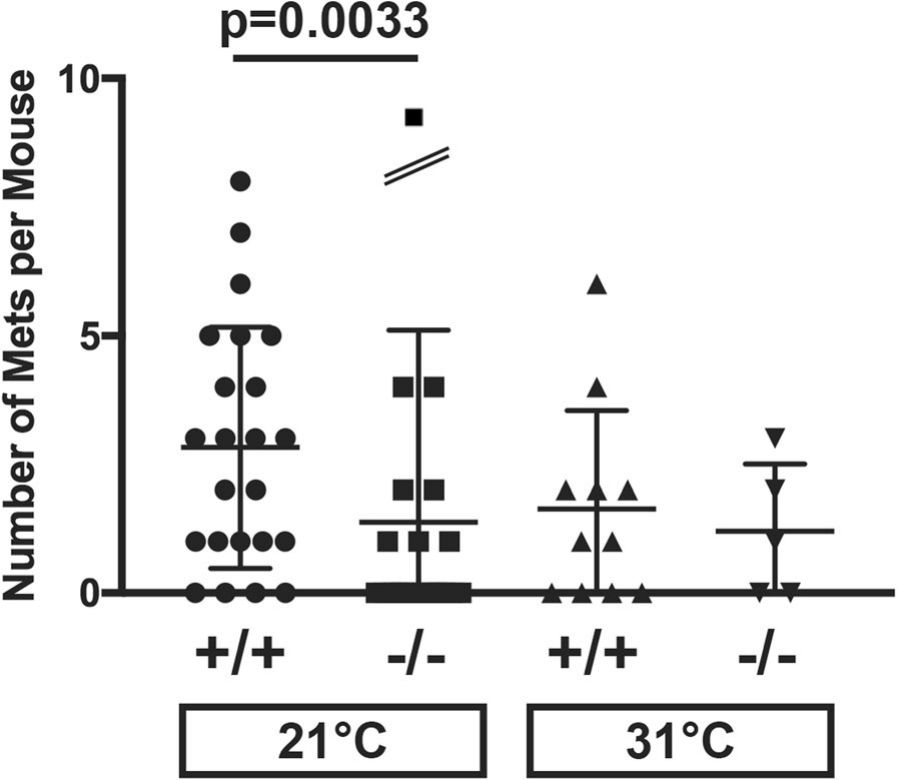
Effect of ambient temperature on metastatic efficiency. A subset of animals was moved to a housing environment with a higher, thermo-neutral temperature of approximately 31 °C, 2 weeks prior to inoculation and maintained at that temperature throughout the duration of the experiment. The 4T1 tumor cells (1 × 10^7^ tumor cells in 10 μL serum-free DMEM) were then injected into the fat pads of Sdc1+/+ and Sdc1−/− mice. The animals were sacrificed 30 days after tumor cell inoculation and lung sections were examined for metastases. Shown are numbers of metastases per mouse (one outlier data point of 18 metastases per mouse in Sdc1−/−; 21 °C group is off scale but is included in mean and SD calculation)

Prior work has shown that under typical, mandated animal housing conditions (20–24 °C), Sdc1-deficient mice are susceptible to cold stress, which is linked to a reduced intradermal fat layer and results in the activation of thermogenesis and subsequent development of a β-adrenergic environment [38, 39]. These stress responses are relieved when Sdc1−/− animals are transferred to thermo-neutral (30–33 °C) temperatures. This result and the prior observation by Kokolus et al. [17] that ambient temperature influences tumorigenesis, tumor growth and metastasis, persuaded us to repeat the 4T1 fat pad injection experiment in mice subjected to higher ambient temperature; i.e., in thermo-neutral conditions. At a housing temperature of 31 °C, metastatic efficiency was indistinguishable between Sdc1+/+ and Sdc1−/− animals (Fig. 5). This suggests that the metastasis-promoting or permissive effect of host Sdc1 requires a sub-thermo-neutral environment.

Since Kokolus and coworkers had also reported correlation between housing temperature and intratumoral CD8+ T cells [17], we measured T cell numbers in our fat pad model. Abundant CD4 and CD8 T lymphocytes were identified in 4T1 lung metastases (Additional file 4: Figure S4 A and B). In larger metastases, these tumor-infiltrating lymphocytes were located primarily in the tumor periphery (not shown). Consistent with the findings by Kokolus et al., intratumoral CD8+ T cell numbers were increased in mice housed at 31 °C compared to 21 °C (Additional file 4: Figure S4 D). In contrast to the Kokolus study, however, we also observed elevated intratumoral CD4+ T cells in mice housed at the higher temperature of 31 °C (Additional file 4: Figure S4 C). Because of the observed effect of housing temperature on intratumoral T cells and on Sdc1-mediated metastatic efficiency, we examined a possible relationship between Sdc1 deficiency and intratumoral T cells. The Sdc1 genotype was not significantly associated with intratumoral CD4+ or CD8+ T cell numbers at either housing temperature (Additional file 4: Figure S4 E and F). In normal lung tissue, CD4+ and CD8+ cell numbers correlated with neither housing temperature nor Sdc1 status (Additional file 4: Figure S4G, H).

## Discussion

Here we show that host Sdc1 is required for efficient metastasis of mammary carcinoma cells and that the HSPG acts by enhancing the outgrowth of metastatic lesions. The host Sdc1 effect is lost when the animals are placed in thermo-neutral housing conditions.

These observations describe a new pathway by which stromal cell-derived Sdc1 can drive cancer progression by stimulating proliferation of disseminated carcinoma cells at distant organ sites. This is relevant because it ascribes a role to Sdc1 at a critical transition step during the natural history of breast cancer. Local disease is typically controlled with surgical and radiation therapy. However, at the time of diagnosis, carcinoma cells may already have disseminated to distant organ sites, where they may lie dormant for many years. The mechanisms that govern escape from dormancy and outgrowth into clinically apparent metastatic lesions are unknown but are thought to rely on microenvironmental cues from the metastatic niche.

Co-localization studies identified Sdc1 expression in intratumoral fibroblasts within the metastatic niche and failed to detect Sdc1 in endothelial cells or leucocytes, pointing to metastasis-associated fibroblasts as the key cell type, regulating outgrowth. This is consistent with our understanding of stromal Sdc1 activity in primary breast carcinomas. However, airway epithelial cells also express Sdc1 and it is possible that epithelial Sdc1 is responsible for or contributes to metastatic outgrowth in the lung microenvironment. We also cannot rule out the possibility that low levels of Sdc1 expression in cell types other than stromal fibroblasts or airway epithelial cells trigger pathways that stimulate disseminated carcinoma cell proliferation.

The exact mechanism of Sdc1-stimulated metastatic outgrowth is uncertain. Judging from the decreased Ki67 proliferation index seen in Sdc1−/− mice, host Sdc1 stimulates proliferation of disseminated carcinoma cells. In 2D and in 3D co-culture models, fibroblast-derived Sdc1 promotes breast carcinoma cell proliferation via paracrine pathways [6, 21]. In these models, paracrine growth stimulation requires proteolytic cleavage of the Sdc1 core protein resulting in shedding of the Sdc1 ectodomain into the pericellular space. Prior work from several groups including ours revealed matrix metalloproteinase (MMP)14 (aka MT1-MMP) as the obligatory “sheddase” [37, 40]. Other investigators identified heparanase combined with MMP9 as a critical enzyme involved in Sdc1 shedding [41]. In our previously published in vitro model, fibroblast growth factor 2 (FGF2) and stroma-derived factor 1 (SDF-1) complete the paracrine signaling loop that begins with the induction of Sdc1 expression in stromal fibroblasts [21]. Whether or not Sdc1 shedding plays a role in the lung microenvironment in vivo is currently unknown. Sdc1 and its intracellular adapter syntenin are also key molecules in the generation of extracellular vesicles of the exosome class [42]. Furthermore, Sdc1 regulates exosome cargo composition [43]. Since exosomes participate in tumor cell-stroma interactions and exosomes have been shown to prepare the pre-metastatic niche [44], it is conceivable that host Sdc1 stimulates metastasis by modulating exosome production or loading.

Although our results point to an Sdc1 effect on metastatic outgrowth, we cannot rule out the possibility that host Sdc1 levels affect tumor cell extravasation. Götte et al. have shown that Sdc1 deficiency increases the adhesion of leucocytes to retinal endothelium [13]. Any role of endothelial Sdc1 in tumor cell extravasation is speculative at this point.

Sdc1 expression in fibroblasts also leads to the production of ECM with an aligned fiber architecture that is permissive to the directionally persistent migration and invasion of carcinoma cells [23]. ECM fiber alignment in vitro requires activation of the αvβ3 integrin [45]. Beauvais and coworkers have shown that clustering of Sdc1 on the cell surface results in the assembly of a trimeric complex that also contains an αv containing integrin and insulin-like growth factor 1 receptor (IGF1R) [11]. Ligand-independent activation of the IGF1R triggers inside-out activation of αvβ3, which could execute ECM fiber alignment. A migration and invasion-permissive microenvironment may enable carcinoma cells to escape microenvironmental niches that suppress growth of disseminated carcinoma cells. Ghajar and colleagues have described a perivascular niche in the lung that traps disseminated mammary carcinoma cells in a dormant state and ascribed a dormancy-inducing activity to endothelial-derived thrombospondin-1 [46].

The dependence of the Sdc1-induced metastasis-promoting effect on sub-thermo-neutral ambient temperatures is intriguing. Relief of cold stress does not readily explain the observation since Sdc1−/− animals in sub-thermo-neutral temperature (i.e. typical, mandated housing temperature) are the only ones in this experiment that experience significant cold stress and their metastasis rate does not change when moved to the higher ambient temperature. Kokolus and co-workers report that raising the temperature to thermo-neutral conditions increases CD8+ T-cells number and activity in the primary tumors, while decreasing T-helper cells [17]. In our study, no significant difference was identified in CD4+ or CD8+ lymphocytes between Sdc1+/+ and Sdc1−/− animals. Therefore, it is unlikely that T cells are responsible for mediating the effect of host Sdc1 on metastasis but we cannot entirely rule out differences in lymphocyte activity.

Targeting disseminated breast carcinoma cells during the long period of dormancy or preventing escape from dormancy is a promising therapeutic goal. Sdc1-mediated outgrowth of metastatic lesions may be targetable by interfering with Sdc1 core protein interactions using peptide competitors [47] or by blocking other molecules associated with the Sdc1 pathway such as integrin cell adhesion receptors or receptor tyrosine kinases like IGF1R. However, any therapeutic intervention will require a detailed understanding of the mechanism of Sdc1 action in the metastatic microenvironment.

## Conclusions

In summary, we show that Sdc1 expression is induced in stromal fibroblasts in the lung metastatic microenvironment and that host Sdc1 is required for efficient outgrowth of mammary carcinoma metastases. In thermo-neutral (higher temperature of 31 °C) ambient housing conditions, Sdc1 deficiency in the host had no impact on metastasis, suggesting that the Sdc1 effect is temperature-sensitive and likely dependent on mild cold stress. These observations assign an important role to Sdc1 during the late stages of the metastatic cascade, the molecular mechanism of which requires further study.

## Additional files

Additional file 1: **Figure S1.** Effect of host Sdc1 on metastatic efficiency of E0771 mouse mammary carcinoma cells. E0771 tumor cells (1 × 10^7^ cells in 10 μL) were injected into the exposed 4th mammary gland as described in “Methods” and mice were killed after 30 days. (A) Metastatic lesion in lung (original magnification × 400; scale bar indicates 100 μm; M, metastasis; L, lung). (B) Number of metastatic lesions per mouse. Metastases were counted on single histologic sections of both lungs. (C) Metastatic tumor burden expressed as percent lung tissue occupied by metastatic lesions. (D) Average area of metastatic lesions expressed in pixels as measured on histologic sections. (TIF 1153 kb)

Additional file 2: **Figure S2.** Characterization of 4T1 primary fat pad tumors. The 4T1 tumor cells (1 × 10^7^ cells in 10 μL) were injected into the exposed 4th mammary gland and mice were killed after 30 days. (A) Hematoxylin and eosin (H&E) stained sections of tumors in fat pad. Carcinoma cells infiltrate adipose tissue (Ad), which contains benign mammary duct (Duct). Scatter plot graph indicates wet weights of tumors excised from Sdc1+/+ and Sdc1−/− mice. (B) Immunohistochemical (IHC) labeling for proliferation marker Ki67. Graph compares Ki67 labeling index between animal genotypes. (C) IHC labeling for endothelial cell marker CD31. Graph compares CD31-positive area between animal genotypes. (D) IHC labeling for alpha smooth muscle actin (αSMA). Graph compares number of αSMA-positive cell clusters between animal genotypes. (E) IHC labeling for macrophage marker F4/80. Graph compares density of F4/80-positive macrophages between animal genotypes. (F) Tumor border imaged by second harmonic generation (SHG) microscopy. White structures indicate fibrillar collagen. Graph compares mean collagen fiber angles relative to tumor boundary between animal genotypes. (TIF 7079 kb)

Additional file 3: **Figure S3.** Effect of host Sdc1 on later stages of E0771 carcinoma cell metastasis. E0771 tumor cells (1 × 10^5^ tumor cells in 100 μL) were injected into the tail veins of C57BL/6 mice, which were killed 15 days later. (A) Metastasis growing around pulmonary vessel (magnification ×400; scale bar indicates 100 μm; V, vessel). (B) Number of metastatic lesions per mouse. Metastases were counted on single histologic sections of both lungs. (C) Metastatic tumor burden expressed as percent lung tissue involved by metastatic lesions. (D) Average area of metastatic lesions expressed in pixels as measured on histologic sections. (TIF 880 kb)

Additional file 4: **Figure S4.** Effect of housing temperature and host Sdc1 on T cells within lung metastases. A subset of animals was moved to a housing environment with a thermo-neutral temperature of approximately 31 °C, 2 weeks prior to inoculation and maintained at that temperature throughout the duration of the experiment. The 4T1 mouse mammary carcinoma cells were inoculated into the mammary fat pad as described. Mice were killed after 30 days and sections of lung tissue were labeled with antibodies to CD4 and CD8. CD4+ and CD8+ intratumoral and normal lung lymphocytes were counted as described in “Methods”. (A, B) Photomicrographs of adjacent sections of small lung metastasis (M) next to vessel (V) labeled with antibodies to CD4 (A) and CD8 (B) (original magnification × 400). (C, D) Density of intratumoral lymphocytes in mice segregated by housing temperature expressed as number of cells per megapixel (MP) of metastasis tissue. (E, F) Density of intratumoral lymphocytes in mice segregated by housing temperature and Sdc1 genotype (same dataset as in C, D). (G, H) Density of lymphocytes in normal lung tissue at distance from any metastases. (TIF 2897 kb)

## Abbreviations

αSMA: Alpha smooth muscle actin
DAPI: 4′,6-Diamidino-2-phenylindole
DMEM: Dulbecco’s modified Eagle’s medium
ER-TR7: Fibroblast antibody
FBS: Fetal bovine serum
H&E: Hematoxylin and eosin
HS: Heparan sulfate
HSPG: Heparan sulfate proteoglycan
IGF1R: Insulin-like growth factor 1 receptor
MMP: Matrix metalloproteinase
NIH: National Institutes of Health
RTK: Receptor tyrosine kinase
Sdc1: Syndecan-1
SHG: Second harmonic generation
