# Supplementary figures and images for: Syndecan-1 induction in lung microenvironment supports the establishment of breast tumor metastases
Source: Breast Cancer Res. 2018 Jul 5;20:66. doi: 10.1186/s13058-018-0995-x (PMC6034333; doi:10.1186/s13058-018-0995-x)

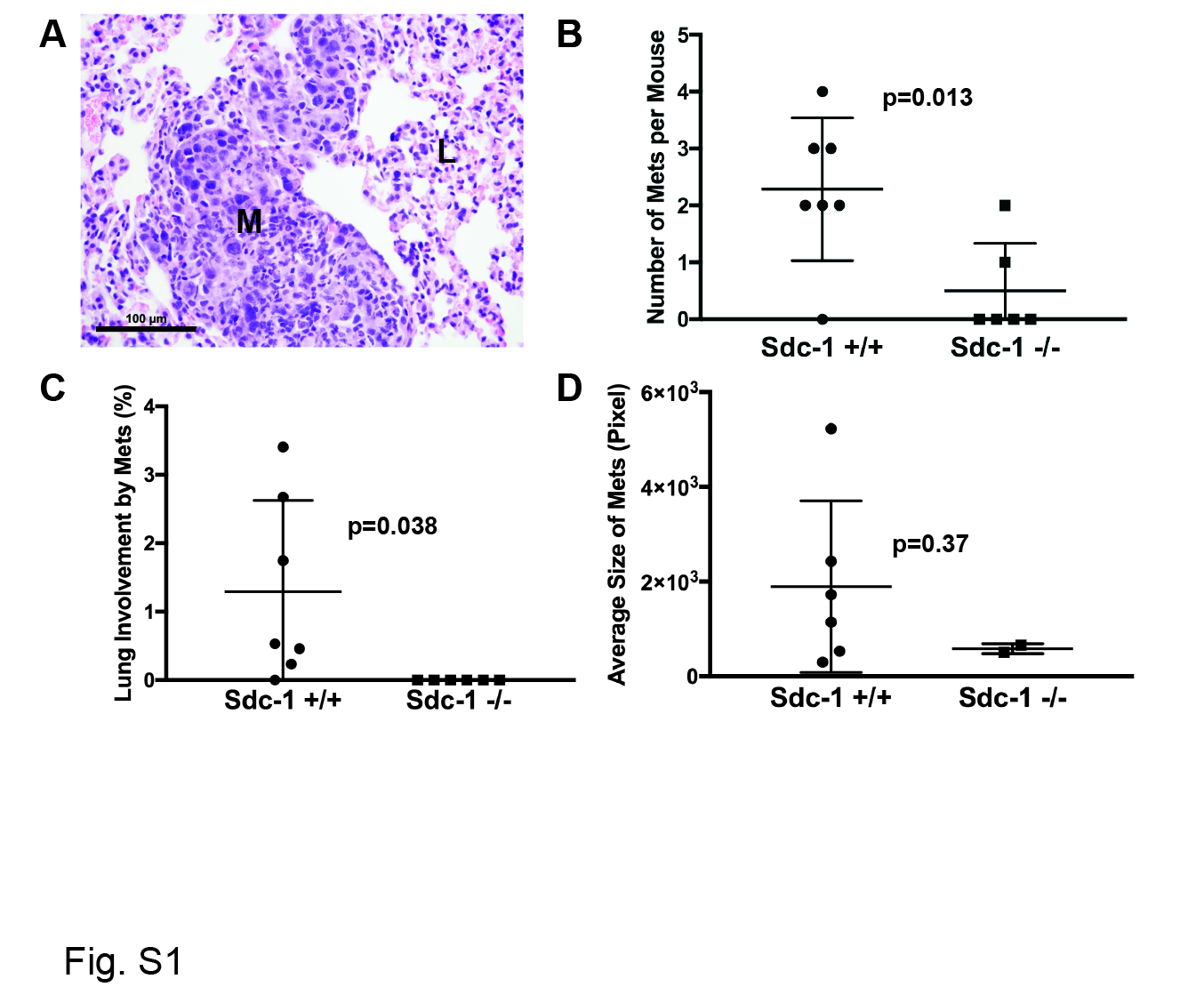

Supplement: Supplementary file 1 — Figure S1. Effect of host Sdc1 on metastatic efficiency of E0771 mouse mammary carcinoma cells. E0771 tumor cells (1 × 10^7^ cells in 10 μL) were injected into the exposed 4th mammary gland as described in “Methods” and mice were killed after 30 days. (A) Metastatic lesion in lung (original magnification × 400; scale bar indicates 100 μm; M, metastasis; L, lung). (B) Number of metastatic lesions per mouse. Metastases were counted on single histologic sections of both lungs. (C) Metastatic tumor burden expressed as percent lung tissue occupied by metastatic lesions. (D) Average area of metastatic lesions expressed in pixels as measured on histologic sections. (TIF 1153 kb) [file 13058_2018_995_MOESM1_ESM.tif]

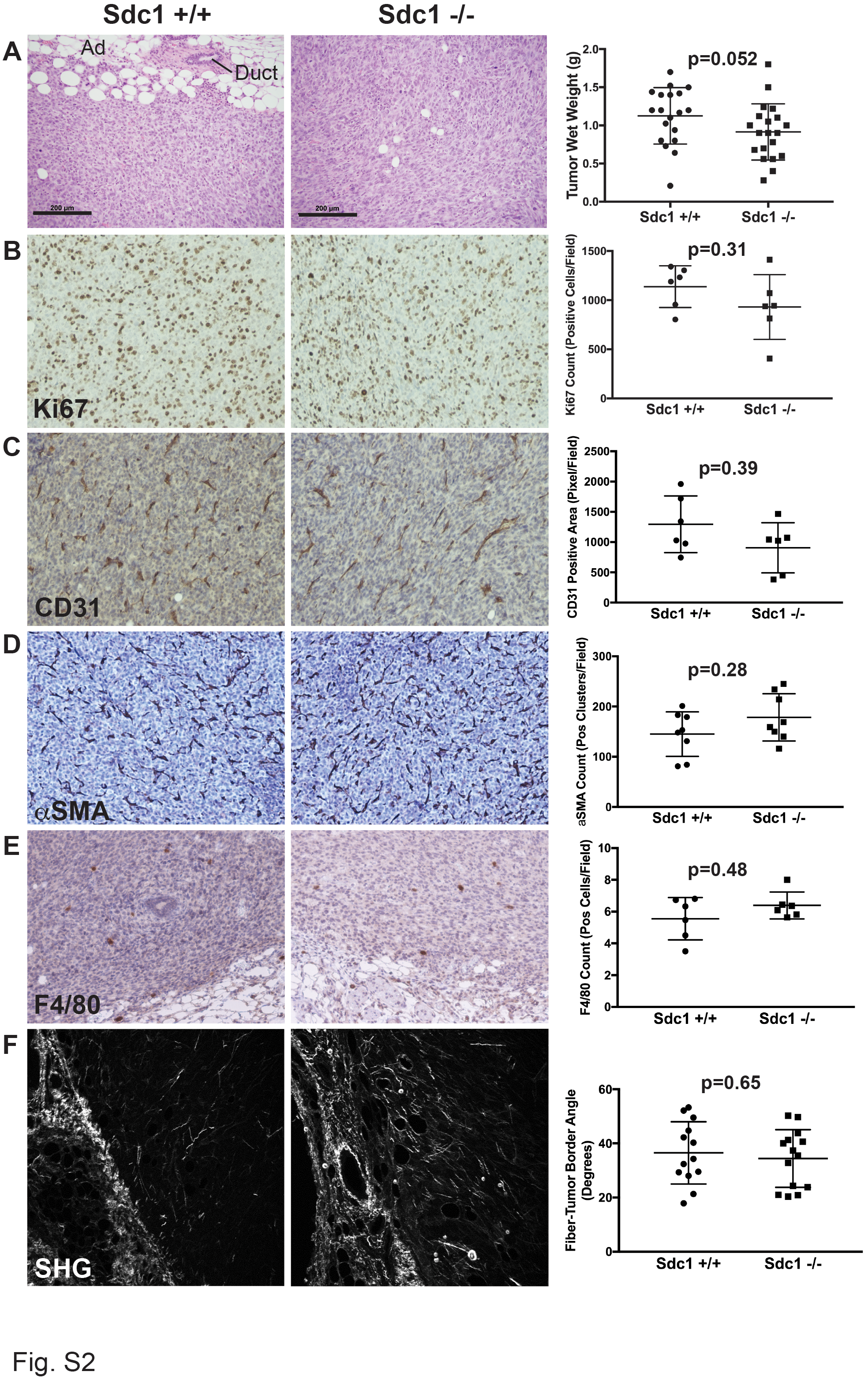

Supplement: Supplementary file 2 — Figure S2. Characterization of 4T1 primary fat pad tumors. The 4T1 tumor cells (1 × 10^7^ cells in 10 μL) were injected into the exposed 4th mammary gland and mice were killed after 30 days. (A) Hematoxylin and eosin (H&E) stained sections of tumors in fat pad. Carcinoma cells infiltrate adipose tissue (Ad), which contains benign mammary duct (Duct). Scatter plot graph indicates wet weights of tumors excised from Sdc1+/+ and Sdc1−/− mice. (B) Immunohistochemical (IHC) labeling for proliferation marker Ki67. Graph compares Ki67 labeling index between animal genotypes. (C) IHC labeling for endothelial cell marker CD31. Graph compares CD31-positive area between animal genotypes. (D) IHC labeling for alpha smooth muscle actin (αSMA). Graph compares number of αSMA-positive cell clusters between animal genotypes. (E) IHC labeling for macrophage marker F4/80. Graph compares density of F4/80-positive macrophages between animal genotypes. (F) Tumor border imaged by second harmonic generation (SHG) microscopy. White structures indicate fibrillar collagen. Graph compares mean collagen fiber angles relative to tumor boundary between animal genotypes. (TIF 7079 kb) [file 13058_2018_995_MOESM2_ESM.tif]

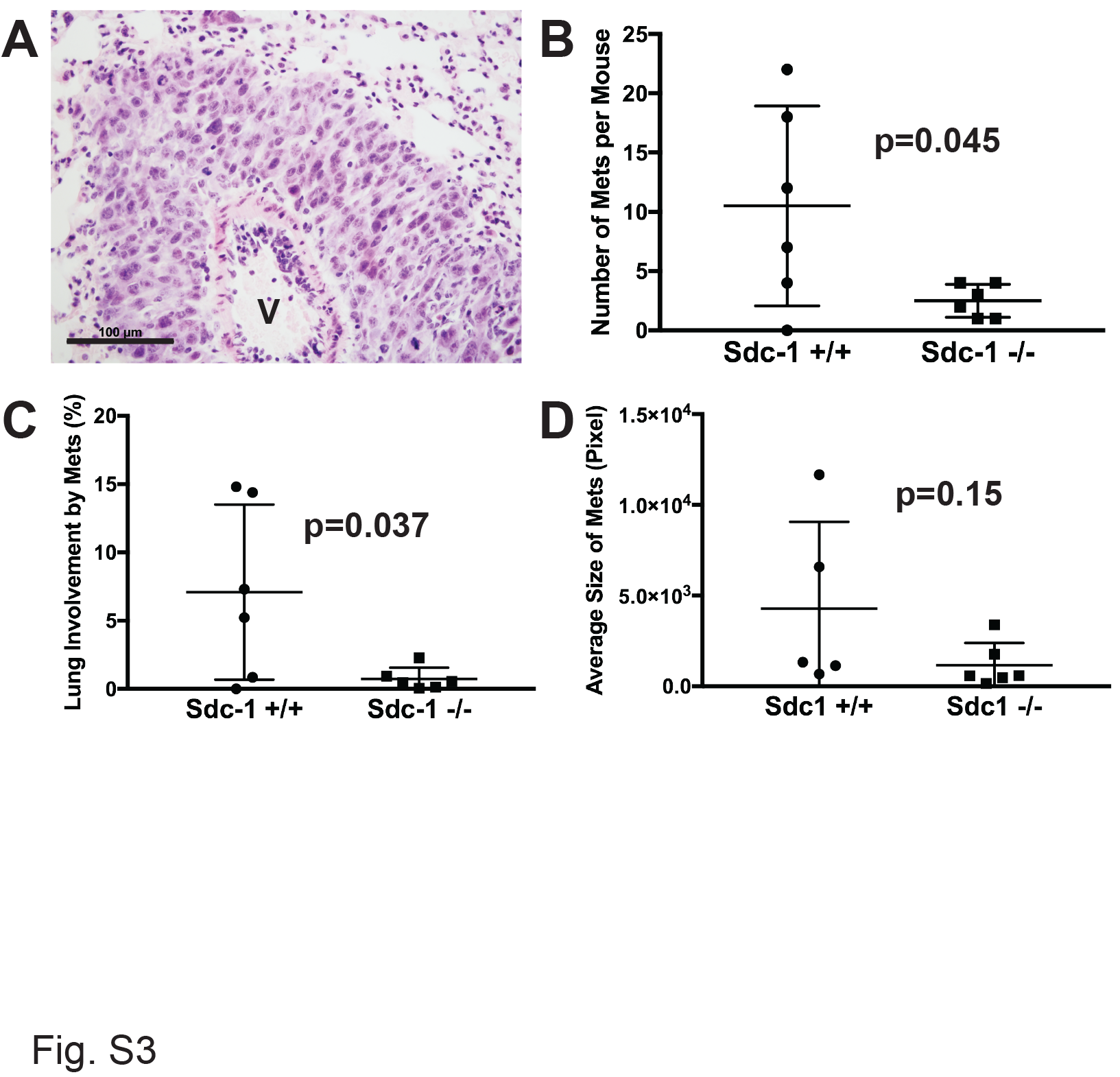

Supplement: Supplementary file 3 — Figure S3. Effect of host Sdc1 on later stages of E0771 carcinoma cell metastasis. E0771 tumor cells (1 × 10^5^ tumor cells in 100 μL) were injected into the tail veins of C57BL/6 mice, which were killed 15 days later. (A) Metastasis growing around pulmonary vessel (magnification ×400; scale bar indicates 100 μm; V, vessel). (B) Number of metastatic lesions per mouse. Metastases were counted on single histologic sections of both lungs. (C) Metastatic tumor burden expressed as percent lung tissue involved by metastatic lesions. (D) Average area of metastatic lesions expressed in pixels as measured on histologic sections. (TIF 880 kb) [file 13058_2018_995_MOESM3_ESM.tif]

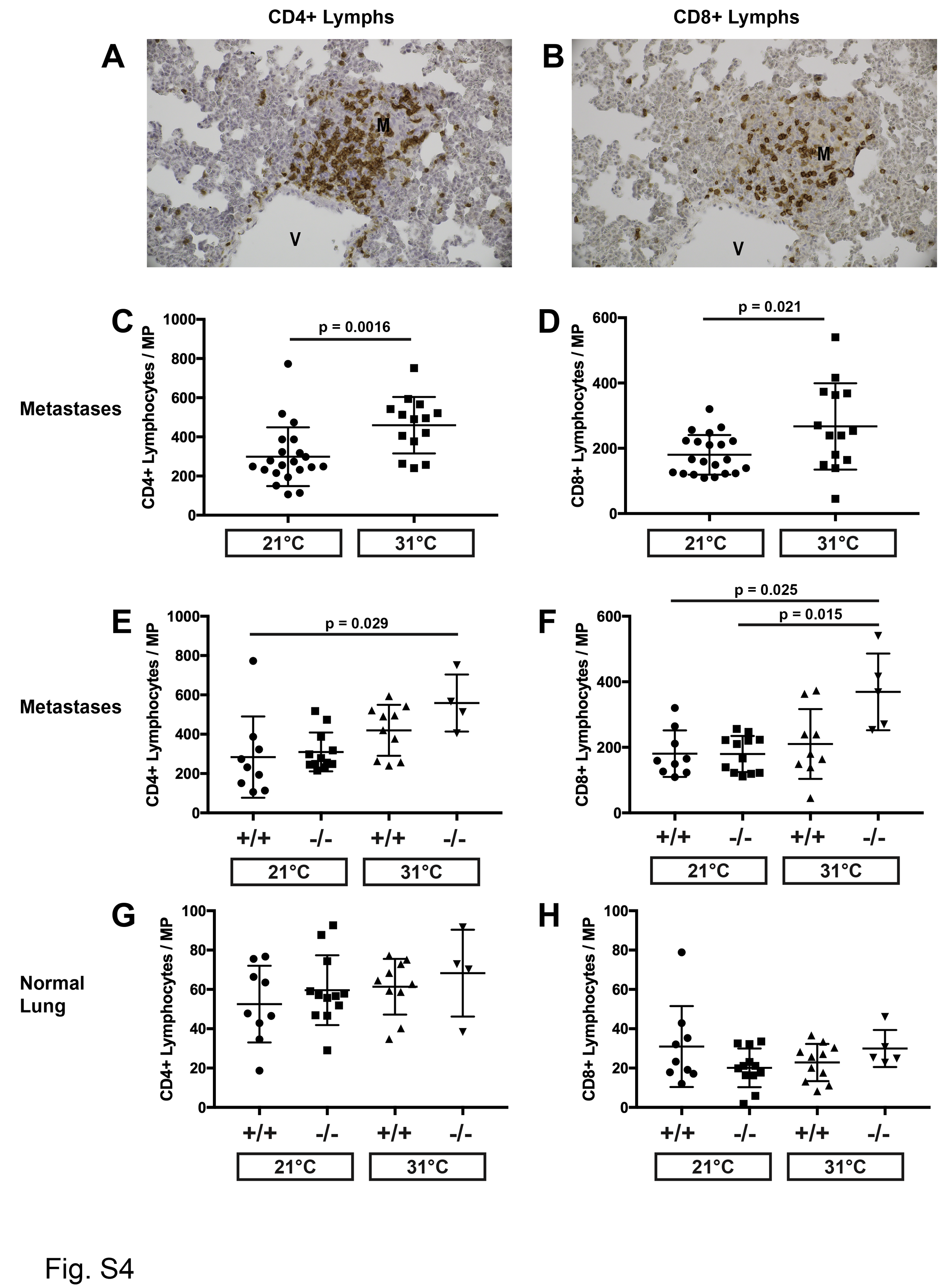

Supplement: Supplementary file 4 — Figure S4. Effect of housing temperature and host Sdc1 on T cells within lung metastases. A subset of animals was moved to a housing environment with a thermo-neutral temperature of approximately 31 °C, 2 weeks prior to inoculation and maintained at that temperature throughout the duration of the experiment. The 4T1 mouse mammary carcinoma cells were inoculated into the mammary fat pad as described. Mice were killed after 30 days and sections of lung tissue were labeled with antibodies to CD4 and CD8. CD4+ and CD8+ intratumoral and normal lung lymphocytes were counted as described in “Methods”. (A, B) Photomicrographs of adjacent sections of small lung metastasis (M) next to vessel (V) labeled with antibodies to CD4 (A) and CD8 (B) (original magnification × 400). (C, D) Density of intratumoral lymphocytes in mice segregated by housing temperature expressed as number of cells per megapixel (MP) of metastasis tissue. (E, F) Density of intratumoral lymphocytes in mice segregated by housing temperature and Sdc1 genotype (same dataset as in C, D). (G, H) Density of lymphocytes in normal lung tissue at distance from any metastases. (TIF 2897 kb) [file 13058_2018_995_MOESM4_ESM.tif]
